# Diet, Physical Activity, Obesity, and Breastfeeding: How French People Perceive Factors Associated with Cancer Risk

**DOI:** 10.3390/nu11102491

**Published:** 2019-10-16

**Authors:** Kristopher Lamore, Pauline Ducrot, Paule Latino-Martel, Marion Soler, Jérôme Foucaud

**Affiliations:** 1 Institut National du Cancer (INCa), 52 avenue André Morizet, 92513 Boulogne-Billancourt, France; kristopher.lamore@parisdescartes.fr (K.L.); msoler@institutcancer.fr (M.S.); 2 Université de Paris, LPPS, F-92100 Boulogne-Billancourt, France; 3 Santé publique France, 12 rue du Val d’Osne, 94415 Saint-Maurice, France; pauline.ducrot@santepubliquefrance.fr; 4 Nutritional Epidemiology Research Team (EREN), Centre of Research in Epidemiology and Statistics (CRESS), Inserm U1153, Inra U1125, Cnam, Paris 13 University, 74 rue Marcel Cachin, F-93017 Bobigny, France; paule.latino-martel@inra.fr; 5 French network for Nutrition And Cancer Research (NACRe network); Bâtiment 400, 78352 Jouy-en-Josas, France; 6 Laboratoire Éducation et Pratiques de Santé (LEPS EA 3412), Université Paris 13—UFR SMBH, 93017 Bobigny, France

**Keywords:** cancer, breastfeeding, cancer barometer, diet, nutrition, obesity, physical activity, population-based study, risk factor

## Abstract

The French Cancer Barometer, a population-based-survey, is carried out every five years and is, to date, one of the few national studies conducted to investigate individual perception linked to cancer risk factors. The aims of the present study were to describe the perceptions of the French population in 2015 and to assess their evolution over a 5-year period (2010–2015). The French Cancer Barometer surveyed a randomly selected sample of participants aged 15–75 years (*n* = 3345 in 2010) and 15–85 years (*n* = 3764 in 2015), representative of the French population. Questions were on perception of diet, physical activity, obesity, and breastfeeding as cancer risk factors. In 2015, nutritional/activity factors were perceived as having an important role in cancer development for the majority of those surveyed (diet (90.8%), obesity (76%), and physical activity (70%)) with the exception being breastfeeding (34%). However, there was a moderate perception of the risks of red meat (43%) and salt or salty food (55%) consumption. Perceptions of nutritional risk factors were mostly associated with age and education level. Interestingly, there was a greater perception of nutritional risk factors in 2015 compared to 2010, and the participants’ opinions were also stronger. Efforts should be made on individuals with lower educational level and to promote the beneficial effects of breastfeeding. However, to impact food behavior, measures are needed at the environmental level and not only at the individual one.

## 1. Introduction

Cancerous disease is always caused by genetic mutations which create a cell division defect and trigger uncontrolled runaway [1]. Apart from the hereditary predisposition identified, a majority of cancers can be attributed to exogenous factors [2]. Even if we do not yet know why mutations occur in one individual and not another, we know that cancer is generally related to several interacting factors [1,2,3,4]. Different factors are known to increase or lower cancer risk [2,3,4], such as age [3], tobacco use [2,3], alcohol consumption [2,3,4], or dietary habits [4]. Prevention strategies have thus been developed and implemented by health agencies and ministries worldwide to raise public awareness on cancer, with the aim of reducing the number of new cases [3,5,6,7,8]. For example, television and poster campaigns encouraging the consumption of five portions of fruit and vegetables per day or informing about the risks of tobacco use are broadcast frequently in European and North American countries. Individuals’ perceptions, attitudes, and behaviors need to be evaluated, frequently and in each country, to adapt these prevention strategies [9,10]. As such, in France, the Cancer Barometer [11,12,13], a population-based-survey, is carried out every five years. In the present study, we focused specifically on the perceptions of the French population regarding the links between nutritional factors and cancer risk. In order to improve prevention strategies, our aims were: (1) to describe the perceptions of the French population in 2015, (2) to identify lesser perceived links between nutritional factors and cancer risk, and (3) to assess the evolution over time (2010–2015) of the French population’s perceptions of cancer risks.

### 1.1. Cancer Statistics

Nearly one in two people will get cancer at some point in their lifetime, as highlighted in studies conducted in Canada, the United States, and the United Kingdom [14,15,16]. In 2018, around 18 million people were diagnosed with cancer [17]. This figure has been increasing steadily over the last 10 years worldwide, with a 33% increase in the number of cases between 2005 and 2015 [18]. Cancer is also known to be one of the leading causes of death in the world, with 9.6 million deaths from the disease in 2018 [2]. In 2018 in France, nearly 382,000 people were diagnosed with cancer, and 157,400 died of cancer [19]. In recent decades, epidemiological studies have thus been conducted to identify cancer risk factors and to implement strategies to reduce cancer incidence worldwide.

### 1.2. Cancer and Risk Factors

Multiple risk factors exist for different cancer sites [2,3,4]. These are divided into endogenous and exogenous factors. Endogenous factors are linked to the individual, such as age and genetic factors [2,3]. Cancer risk increases with age: the median age for cancer diagnosis is 66 years, with one-quarter of new diagnoses occurring between 65 and 74 years [3]. Exogenous factors refer to the environment and people’s lifestyles. Environmental factors include exposure to chemical factors (e.g., arsenic, nickel), radiation and pollution (e.g., radon, ultraviolet radiation), and viral infections (e.g., papilloma virus, hepatitis C) [2,3]. Lifestyle factors include for example dietary habits, alcohol consumption, tobacco use, and exposure to environmental tobacco smoke. In addition, overall health (e.g., overweight) may also play a role in cancer development [2,3,4]. All combined exogenous factors are responsible for up to 55% of cancer cases [20,21,22,23].

### 1.3. Cancer and Nutrition

Nutrition (i.e., an adequate and balanced diet), physical activity, and weight status (i.e., underweight, normal weight, overweight and obesity) are interrelated [24]. In the case of cancer, international expert groups frequently assess the level of evidence for these factors. Thus, guidelines evolve frequently according to the reports published by the World Cancer Research Fund (WCRF) and the American Institute for Cancer Research (AICR). In 2018, their third report [4] listed the factors increasing cancer risk with a probable or convincing level of evidence: presence of overweight and obesity (i.e., having a body mass index between 25 and 30 for overweight, or greater than 30 for obesity), and excessive consumption of red meat and processed meat (including cold meats). Regarding salt and salty foods, the WCRF and AICR report recommend not consuming salt-preserved, salted and salty foods. Cantonese-style salted fish and foods preserved by salting consumption have been highlighted as probable cancer risk factors. Factors lowering cancer risk are also listed: consumption of wholegrain products, fruit and vegetables, fiber, dairy products (e.g., milk), and coffee, as well as regular physical activity and breastfeeding. However, for some nutritional factors (e.g., white meat, fish, and potato consumption), there is no strong evidence regarding their effect on cancer risk. The proportions of cancers attributable to nutritional factors are significant [20,21,22,23]. In France [23], as well as in the United Kingdom [22] and the United States [25], about 18% of all cancers are attributable to nutritional factors. It is therefore important to take action on nutritional factors to reduce cancer incidence.

### 1.4. Improving National Prevention Campaigns

Some population-based studies have shown that compliance with cancer prevention guidelines (with respect to body mass index, physical activity, diet, and alcohol consumption) is associated with a lower risk of cancer [9,10]. Therefore, prevention strategies aimed at disseminating healthy nutritional practices may have a positive impact on cancer development. Studies investigating knowledge, perceptions, attitudes, behaviors and/or practices have thus been conducted internationally to highlight potential barriers to behavioral change [26,27] and adapt national prevention programs [28]. However, studies investigating cancer risk and nutritional factors have focused essentially on subjects’ knowledge and behaviors, with each nutritional factor being studied individually (e.g., subjects’ knowledge of the cancer risks attributed to diet). Furthermore, these studies have focused on specific groups such as athletes [29,30], health professionals [31,32,33], or young people [34]. Research on public awareness of cancer risk factors has thus rarely been conducted and has only briefly explored cancer risks attributed to nutrition [35,36,37,38,39,40]. The Cancer Barometer was thus set up in France to investigate French people’s perceptions, attitudes and behaviors regarding cancer causes [11,12,13].

## 2. Materials and Methods 

### 2.1. Study Desgin

For this population-based study, we used data from the Cancer Barometer conducted in France in 2010 and 2015. This national survey, using computer-assisted telephone interviews, has investigated perceptions and knowledge related to cancer, cancer prevention and cancer risk factors (alcohol, tobacco, and exposure to ultraviolet radiation) since 2005. The questions are consistent throughout the time period; however, further questions about nutritional factors (diet, physical activity, weight status, and breastfeeding) and cancer risks were added in 2010 and asked again in 2015. The survey was conducted in accordance with the Declaration of Helsinki. The complete methodology of the surveys is presented elsewhere [41,42].

### 2.2. Population and Recruitment Method

The study was proposed to people aged between 15–75 years in the 2010 and 2015 Barometers, living in France, speaking fluent French and who had never had a cancer diagnosis. In the 2015 Barometer, subjects aged between 75 and 85 years were also included to be more representative of the French population [43]. To be included in the study, participants needed to have access to a landline or mobile phone.

A list of potentially eligible participants was generated using random digit dialing using number prefixes corresponding to the blocks of numbers assigned by the French regulator. When several people used the same phone number, the Kish method [44] was applied to select the eligible person in the household. This method was used to survey a representative sample of the French population. Before starting recruitment, a pilot study was conducted with 100 and 119 subjects, in 2010 and 2015, respectively, to validate the comprehension of the questionnaire and the participant selection method. The recruitment took place Monday to Saturday from 3 April to 7 August 2010, and from 19 May to 13 October 2015 (with a three-week break in August).

### 2.3. Procedure

An information letter was first sent to all the potentially eligible participants of the survey. They were then contacted by phone and verbal consent was obtained. When the subject was contacted but was unavailable, an appointment was made. When no one answered the phone, at least 40 attempts were made to carry out a survey (at different times of the day and different days of the week). When subjects were contacted and available, inclusion criteria were verified before asking the closed and multiple-choice questions from the list. Computer-Assisted Telephone Interviewing (CATI) Software was used by the interviewers as a phone assistance device to manage phone calls, appointments and the progression of the survey. The subjects’ responses were anonymous and self-reported. The study was conducted in accordance with French Data Protection Commission (CNIL) guidelines and carried out with assistance from the *BVA Group* who are accredited to conduct population-based phone surveys.

### 2.4. Survey

The 2010 and 2015 surveys started with sociodemographic questions and general information-related questions for the participants. The following information was collected: sex, age, city, education level, current or previous employment, and monthly family income in Euros per consumption unit (CU; i.e., one CU is attributed for the first adult in the household, 0.5 for other persons aged 14 or older, and 0.3 for children under 14 years old). In the analysis, three income ranges were considered: €0–1100/CU, €1100–1800/CU, and >€1800/CU. These ranges are based on the minimum wage and national data on individual salaries, representing three social classes: low income, middle income, and high income. 

Regarding the links between nutritional factors and cancer risk, three main multiple-choice questions were presented:

(1) “Do you think that diet has a ‘very important’, ‘somewhat important’, ‘somewhat unimportant’ or ‘not at all important ’role in cancer development?”.
(2) “In your opinion: frequent consumption of (‘fruit and vegetables’, ‘red meat’, ‘milk’, ‘cold meats’, ‘white meat’, ‘fish’, ‘salt and salty foods’, ‘potatoes’): (‘can lower’, ‘can increase’, or ‘has no influence on’) cancer risk. The question was repeated for participants who responded ‘Don’t know’. If they still could not answer, the response ‘Don’t know’ was recorded. In our survey, participants were surveyed on cold meats (i.e., ham, salami, dry sausage, lomo Serrano, etc.) and not on processed meat in general as eating cold meats is of cultural significance in France.
  Question (2) was adapted for physical activity, overweight and obesity, and breastfeeding (for women only)—“In your opinion, (‘regular physical activity’, ‘overweight & obesity’, ‘breastfeeding’): (‘can lower’, ‘can increase’, or ‘has no influence on’) cancer risk”.
(3) “Do you feel that you are ‘very well, ‘somewhat well’, ‘somewhat poorly’ or ‘very poorly’ informed on the health effects of (‘diet’, ‘physical activity’)?”.

### 2.5. Statistical Analysis

All statistical analyses were conducted using SAS enterprise guide 7.13. First, descriptive analyses were used to describe the participants’ characteristics and perceptions. Proportions were described as ‘very low’ when under 30%, ‘low’ when between 30% and 40%, ‘moderate’ when between 40% and 60%, ‘high’ when between 60% and 70%, and ‘very high’ when higher than 70%. These cut-offs are based on a Gaussian curve, with a theoretical mean of 50, and standard deviation of 10. Chi-squared tests were performed to study differences in sociodemographic variables associated with participants’ perceptions. Finally, logistic regressions were performed to evaluate the associations between sociodemographic variables and the perception of nutritional factors in cancer risk. A stepwise analysis was performed to define the explanatory variables of each regression model. Variables added to the models were: age, sex, monthly family income, education level, occupation, and perceived levels of information on the effects of diet and physical activity on health.

For Question (1) and (2), the responses ‘very important/well and ‘rather important/well’ were combined, as were the responses ‘somewhat unimportant/poorly’ and ‘not at all important/very badly’, to obtain a clear result on the number of participants perceiving the cancer risks linked to nutritional factors and their level of information (well informed versus poorly informed) or not.

## 3. Results

Table 1 presents the complete information on participant recruitment. In total, 25,706 and 69,292 phone numbers were generated in 2010 and 2015. Of these phone numbers, only 9852 and 16,601 (in 2010 and 2015 respectively) were eligible and contacted. Our final sample was composed of 6853 subjects aged between 15 and 75 years (*n* = 3345 in 2010 and *n* = 3508 in 2015). Table 2 presents the demographic and socioeconomic characteristics of the participants. In 2015, 256 participants aged 75 and 85 years were surveyed, but their responses were not included in the analyses performed to compare 2010 and 2015 results.

### 3.1. Perceptions of Nutritional Factors in Cancer Risk in 2015

#### 3.1.1. Perception of Role of DIET in cancer Risk

In 2015, diet was perceived by the participants as having an important role (90.8%) in cancer development. Different sociodemographic factors were associated with this perception, as presented in Table 3. Univariate and multivariate analyses showed differences regarding sex, age, family income, region of residence, and education level. No significant difference was observed as regards the participants’ occupation (not shown). The perception of the impact of diet on cancer risk was significantly greater among women, older people, subjects with higher income and education levels or living outside the Paris region (as shown in the multivariate analyses presented in Table 3). Furthermore, subjects who considered themselves better informed about the effects of diet on health (91.5% versus 89.5% who considered themselves poorly informed, *p* <0.05) and who have had someone close to them diagnosed with cancer reported a greater perception of the effects of diet on cancer development (91.2% versus 86.9%, *p* <0.05) (not tabulated).

The importance of diet in cancer risk varied also regarding the types of food. As presented in Table 4, foods known to be associated with cancer risk were moderately perceived as increasing (42.6% for red meat, 62.2% for cold meats, and 54.6% for salt and salty foods) or lowering cancer risk (58.1% for fruit and vegetables). There was a very low perception of milk consumption as lowering cancer risk (11.8%). For foods not known to be associated with cancer risk, the majority of the participants indicated that they had no influence on cancer risk (64.7% for potatoes and 57.3% for white meat), except for fish consumption where 38.3% stated an increased risk and 39.4% stated that fish consumption has no influence on cancer risk.

The multivariate analysis revealed how food perceptions toward cancer risk varied according to sex, age, family income, education level, occupation, and perceived level of information regarding the effects of diet on health. Four logistic regression models are presented in Table 5 for foods known to be associated with cancer risk. Regarding sex, this factor has a significant impact only for salt and salty food perception: indeed, women had a lower perception of their impact on cancer risk compared to men (odds ratio, OR = 0.9, 95% confidence interval = 07–1.0, *p* < 0.05). Regarding age, the impact of fruit and vegetables, red meat, and cold meat consumption on cancer risk had a greater perception among older people, while the effect of salt and salty foods consumption on health had a greater perception among young people. Regarding education level, participants with higher education levels had a greater perception of the impact of food consumption on cancer risk, except for salt and salty foods, but the differences were not significant. Regarding occupation, red meat was the only food not significantly associated. Finally, the links between cancer risk and fruit and vegetable, red meat, cold meat, and salt and salty food consumption were more perceived by participants with higher family income and higher perceived levels of information regarding the effects of diet on health.

#### 3.1.2. Perception of the Link between Regular Physical Activity and Cancer Risk

Regular physical activity was perceived by 70.0% of the participants as lowering cancer risk (see Table 4). Multivariate analysis revealed how the perception of the impact of frequent physical activity on cancer risk varied according to sex, age, family income, education level, occupation and perceived level of information regarding the effects of physical activity on health (see Table 6). There is significantly greater perception of the effect of regular physical activity on cancer risk among men, young people (15–24 years old), families with higher monthly income (€1101–1800 per CU), those with higher education levels (secondary and university), employees, and participants who considered themselves well-informed about the effects of physical activity on health.

#### 3.1.3. Perception of the Link between Overweight and Obesity and Cancer Risk

Overweight and obesity was perceived by 75.7% of the participants as increasing cancer risk (see Table 4). Multivariate analyses revealed how the perception of the role of overweight and obesity in cancer risk varied according to sex, age, family income, education level, occupation, and perceived level of information regarding the effects of physical activity and diet on health (see Table 6). There was a significantly greater perception of the effect of overweight and obesity on cancer risk among men, young people (15–24 years old), families with higher monthly income (€1101–1800 per CU), those with higher education levels (secondary and university), those with intermediate occupations, and participants who considered themselves well informed about the effects of physical activity and diet on health.

#### 3.1.4. Perception of the Link between Breastfeeding and Cancer Risk

Breastfeeding was perceived by 34.0% of the female participants as lowering mothers’ breast cancer risk (see Table 4). Multivariate analyses revealed how the perception of the impact of breastfeeding on cancer risk varied according to age, family income, education level and occupation (see Table 6). There was a significantly greater perception of the effect of breastfeeding on mothers’ breast cancer risk among older people (over 25 years), women with lower family income (under €1100 per CU, compared to women who did not know or declined to state their family income: OR = 0.5, 95% confidence interval = 03–0.8, *p* <0.01), those with a higher education level (secondary and university), and women from the working class (i.e., laborers), while there was a lower perception of breastfeeding benefits among women who had never worked compared to employees (OR = 0.5, CI = 03–0.8, *p* <0.01).

### 3.2. Evolution of Perception of the Impact of Nutritional Factors on Cancer Risk between 2010 and 2015

**Figure 1:**
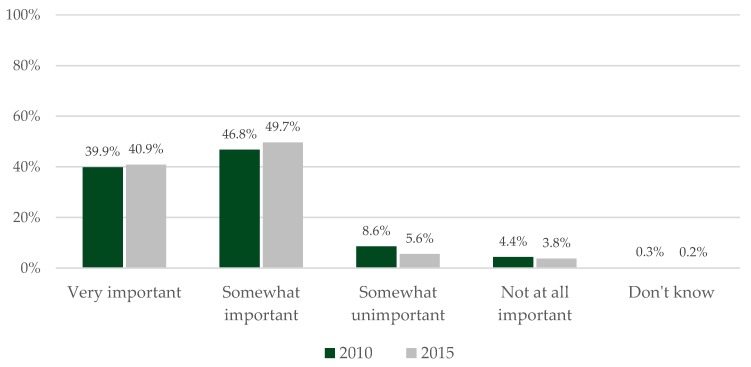
Evolution between 2010 and 2015 of the perceived importance of the role of diet in cancer risk (*p* < 0.001).

There was a greater perception among participants of the role of diet (see Figure 1) and of the different nutritional factors in 2015 compared to 2010 (see Table 7) (χ^2^ = 29.6, degrees of freedom = 4, *p* <0.001). Notably, there was a greater perception of cold meat (+14.4%) and salt and salty food (+18.5%) consumption in 2015 than in 2010.

In 2015, participants had a stronger opinion than in 2010, as significantly fewer responded “Don’t know” to the different questions (see Table 7). However, participants also responded “Has no influence on cancer risk” more frequently in 2015 than in 2010.

## 4. Discussion

There is a greater perception of risk factors linked to nutrition, physical activity, overweight and obesity, and breastfeeding in 2015 than in 2010. This finding is encouraging and highlights that French people are more aware of cancer risks. However, the findings should be interpreted with caution in national surveys comparing two different samples (i.e., the participants surveyed in the second survey are not the same as those in the first survey) as this could lead to misinterpretations [26,27]. Participants surveyed in 2015 had stronger opinions (i.e., less use of the response “Don’t know”) and had a greater perception of the impact of diet (essentially for salt and salty foods, red meat and cold meats), physical activity, and overweight/obesity on cancer risk. However, there was a greater perception of foods for which consumption has a protective effect (i.e., milk and fruit and vegetables) among participants in 2015 than in 2010.

The strengths of the Cancer Barometer lie essentially in two points: (1) a representative sample of the French population is surveyed, and (2) a review is conducted every five years. The findings of this study are thus generalizable to the French population and can be used to adapt cancer prevention programs by improving community-wide interventions. Regarding the exploration of cancer risks attributed to nutritional factors, this is also one of the first studies investigating public awareness on this subject in such detail.

Physical activity, weight status, and diet are perceived by the majority of the participants as having an important role in cancer development. However, when we look at each food group known to be associated with cancer risk [4], the awareness of participants differed. Participants had a good perception of the role of cold meats (increasing risk), according to other findings of the Cancer Barometer [45], but to a lesser extent than those for fruit and vegetables, and salt and salty foods (lowering and increasing risk, respectively). There was little perception of milk consumption as increasing cancer risk. There is a greater perception of cold meat consumption as a cancer risk factor as it is also associated with an increase in other diseases (e.g., diabetes, cardiovascular diseases) [46,47,48]. For fruit and vegetables and salt and salty food, links with cancer risk were not well identified by participants, whereas these food groups are recognized by individuals as good and bad for health, respectively [49,50,51]. In 2007 [52], the influence of milk on the development of cancer was not known, while in 2018 [4], milk consumption was highlighted as lowering cancer risk. A potential explanation of people’s perceptions might be that French prevention campaigns have broadcast messages to encourage or limit the consumption of certain foods but have not mentioned the rationale regarding cancer prevention. It could be of interest for future research to investigate why such differences are observed between food groups and whether raising the perception of the links between food consumption and cancer might encourage people to change their food habits. 

Breastfeeding is ultimately the nutritional factor with the lowest perception as lowering cancer risk. This could be due to a lack of communication in France about the benefits of breastfeeding for women. Breastfeeding is beneficial for the baby (e.g., helps the baby fight off viruses and bacteria), but it also lower women’s cancer risk, risk of osteoporosis, and can be helpful for post-childbirth recovery (e.g., to reduce uterine bleeding) [53]. However, the many health benefits for breastfeeding women seem to be unknown to women [54,55,56]. In addition to a lack of knowledge, breastfeeding is associated with the fear linked with this behavior, demographic factors, social norms, employment, lactation problems, and culture [57,58,59]. In France, women breastfeed their child 17 weeks on average [60], which is less than the international guidelines of 6 months [4]. This finding underlines the need to better understand individuals’ perceptions about breastfeeding in France and to develop national prevention campaigns. Qualitative studies investigating women’s perceptions of the health benefits represented by breastfeeding for both women and their babies need to be conducted first. To our knowledge, studies essentially focus on women’s knowledge of breastfeeding benefits for babies rather than for women.

Perception of the impact of nutritional factors on the onset of cancer is linked with sociodemographic characteristics. In our study, sex, age, education, occupation, and monthly income were frequently associated with participants’ perceptions. For example, there was a lower perception of the benefits of physical activity and the risks of overweight and obesity among participants over 24 years of age, less educated people, and those with low incomes. In France, as in many other countries, the prevalence of overweight and obesity has remained higher among the least educated [61]. It appears that those most affected have a lower perception of the risks that their condition can represent. Thus, it is necessary to continue to provide information on the health benefits of physical activity and the risks represented by overweight/obesity, in particular by indicating the health risks. However, individuals perceptions are not necessarily in keeping with their behavior. Different variables are likely to influence behavioral change in individuals (e.g., level of knowledge, attitudes, influence of the information provider) [62,63,64,65]. Several theories and models have been proposed to help design prevention interventions targeting behavioral change [62,63,64,66,67] and can be used to design new interventions. It seems of interest to conduct national and longitudinal studies to highlight potential barriers to behavioral change [26,27]. 

The first report on ‘Food, nutrition and the prevention of cancer’ was published in 1997 by the WCRF and AICR. This report was subsequently updated in 2007 and in 2018 [4]. The importance of providing national prevention campaigns on this subject appeared after 2007 in France [67], following the publication of the WCRF and AICR reports [4]. National public health policies are different and, thus, we can assume that national campaigns on nutrition and cancer appeared at different times worldwide. Surveys highlighting peoples’ attitudes, knowledge, and/or perceptions should be conducted in each country to develop prevention campaigns adapted to the population. To date, few countries have conducted health surveys or cancer barometers to study populations’ attitudes, knowledge and/or perceptions about cancer risk factors. We found seven studies conducted in Belgium, Ireland, Japan, Oman, Spain, the United Kingdom, and the United States [35,36,37,38,39,40,68]. Cancer risk attributed to nutritional factors is not explored extensively in these studies. For example, in Spain, in 2012, 27% and 54% of the respondents respectively perceived weight status and diet as important risk factors in cancer [38]. In the United States, a national opinion survey conducted in 2017 highlighted that only 31% and 28% of respondents perceived obesity and food choices as cancer risk factors [36]. In comparison to these two national studies, respondents to the French Cancer Barometer, both in 2010 and 2015, appear to be more aware of the impact of nutritional factors on the onset of cancer. However, the investigation of these risk factors remains relatively recent.

International nutritional guidelines for cancer prevention exist [69] and are consistent with guidelines aimed at the prevention of other chronic diseases [70,71]. However, nutritional guidelines for cancer prevention are not clearly integrated into national prevention campaigns. In France, cancer prevention campaigns have, in the past, had a strong or more frequent focus on tobacco, as this is the main factor associated with cancer development [23]. Prevention campaigns concerning nutrition are more focused on the benefits for overall health. These French Barometers highlight the need for national prevention campaigns on the impact of nutritional factors on the onset of cancer to be developed. Following the Cancer Barometer study conducted in 2010 and 2015, national campaigns focusing on nutrition and alcohol were implemented for the first time. Information on cancer is provided on the *French National Cancer Institute* website (www.e-cancer.fr) and updated as needed. Information on nutritional factors, alcohol and tobacco, and cancer risks has also been provided through posters, print media, television and radio each year. For example, in 2016, posters were displayed in pharmacies to raise people’s awareness. In 2019, television commercials were broadcast. Furthermore, the survey’s findings have been made available to French public health agencies such as the *French National Authority for Health*. Unfortunately, we have had no feedback on how public health agencies have used the findings. In the future, we should establish a method to evaluate how useful the findings of the Cancer Barometer are and how they are used by public health agencies.

Due to the importance of demographic factors on individuals’ perceptions, community-wide and health education interventions can be designed to increase knowledge and awareness of those less aware of the cancer risk associated with diet. Proof-of-concept and feasibility studies [72], using qualitative study designs, could be used to develop adapted interventions.

Some limitations need to be pointed out. During the study, a national report on nutrition and cancer risk was published in France [73]. Participants may have consulted this report, which may have influenced their answers. To limit this bias in future studies, researchers should request the number of days/months/years since respondents last perceived information about nutrition and cancer risk or health. Furthermore, it is not always clear if the Cancer Barometer studies investigated perceptions and/or knowledge, as knowledge can influence beliefs and perceptions. The questions asked in this survey also do not allow us to identify the exact reason for the improvement in French people’s awareness of cancer risk. Nevertheless, the findings help identify factors with a lower perception among the population as increasing or lowering cancer risk. In addition, the question related to breastfeeding was only put to women. Future surveys should also survey men as individuals’ behaviors are influenced by those close to them [74,75,76]. Regarding participant recruitment, one limitation can be pointed out. Participants’ ethnic or religious origins were not recorded. In France, it is not ethically possible to record a person’s ethnic or religious origin. However, culture (including ethnicity and religion) can impact peoples’ perceptions [77]. Thus, the participants’ culture should be surveyed in future studies when ethics committees allow such questions. Finally, the methodology of the study can be improved for future surveys (2020 and beyond). The findings of the French Cancer Barometer would gain in robustness if participants’ behaviors and levels of confidence in their responses were assessed. This would make it possible to assess whether people’s behaviors are in line with their perceptions or not, and vice versa, to improve cancer prevention programs.

## 5. Conclusions

These two national surveys at two time points, 2010 and 2015, highlight the need to improve prevention policies concerning cancer risk and nutrition.

People with a lower perception of the links between nutrition and cancer risk are lower socio-economic groups and individuals with lower education levels. Therefore, policies should be designed within a proportionate universalism framework in order to reach the general population (universal) with a proportionate targeting that takes into account the social gradient. Thus, community-wide could be proposed. However, to promote healthy food choices, it is essential that policies target the nutritional environment. Examples of such mesures include levies and taxes, advertising regulation or nutrition labelling (e.g. Nutri-Score adopted in France), which may encourage product reformulation and therefore lead to a food offer improvement. By making healthy food more accessible, affordable and visible, environmental measures are more likely to impact food behavior and to support the information disseminated through prevention campaigns. Regarding breastfeeding, interventions targeting the entire French population seem more appropriate, as there is a low perception of this factor among women as lowering cancer risk and this factor is a social issue. The next French Barometer surveys could be improved with questions assessing the participants’ reasons for their perceptions. 

## Figures and Tables

**Table 1:** Participant recruitment.

	2010	2015
***n* phone numbers generated**	25,706	69,692
Phone number not available ^1^	0	38,204 (54.8%)
Contactable numbers	25,706 (100%)	31,488 (45.2%)
Not eligible according to inclusion criteria	15,854 (61.7%)	1898 (6.0%)
Not contacted ^2^	0	12,989 (41.3%)
***n* eligible phone numbers**	9852	16601
No interview carried out ^3^	576 (5.9%)	2144 (12.9%)
Declined to participate	5270 (53.5%)	9839 (59.3%)
Drop-out ^4^	278 (2.8%)	479 (2.9%)
Participation completed	3728 (37.8%)	4139 (24.9%)
Missing data	383	375
Final sample	3345 (34.0%)	3764 (22.7%)

Legend: ^1^ The phone number generated was not attributed; ^2^ No one was contacted after 40 attempts; ^3^ In some cases, an appointment was made but the subject was no longer contactable; ^4^ The participation was discontinued during the survey and not completed.

**Table 2:** Participant characteristics for 2010 and 2015 Cancer Barometer surveys.

Variables	2010 (*n* = 3345)	2015 (*n* = 3508)	*p*-Value for χ^2^ Test
	%	%	
**Sex**			***
Male	43.8	49.0	
Female	56.2	51.0	
**Age**			***
15–34 years	30.6	32.1	
35–44 years	20.8	17.5	
45–54 years	18.7	17.7	
55–64 years	19.2	15.9	
65–75 years	10.7	16.8	
**Monthly income (€/CU) ^1^**			***
€0–1100	27.4	31.6	
€1101–1800	34.4	34.7	
> €1800	30.0	25.1	
Don’t know/Declined to answer	8.2	8.6	
**Education level**			***
<High school	46.4	53.0	
High school	19.7	19.1	
University level	33.9	27.9	
**Occupation ^2^**			***
Employee	22.8	25.6	
Laborer	17.4	23.8	
Tradesperson, storekeeper, self-employed worker, or farmer	6.1	7.2	
Intermediate occupations	28.3	19.2	
Higher occupations	21.0	19.1	
Other non-workers	4.4	5.3	

Legend: *** = *p* <0.001. ^1^ CU = consumption unit. One CU is attributed for the first adult in the household, 0.5 for other persons aged 14 or older, and 0.3 for children under 14 years. ^2^ The occupations listed are commonly used in France. “Employee” refers to employees with limited responsibilities such as administrative employees, or staff in direct contact with customers. “Laborer” refers to workers at a factory, drivers, or handling workers. “Intermediate occupations” refers to employees with intermediate responsibilities, such as nurses, schoolteachers or social workers. “Higher occupations” refers to employees with senior responsibilities, such as professors, engineers, physicians, or department directors.

**Table 3:** Sociodemographic factors associated with the perception of diet as an important factor in cancer development in 2015 (*n* = 3413).

Variables	Univariate analysis			Multivariate Analysis			
	*n*	%	*p*-Value for χ^2^ Test	OR	95% CI		*p*-Value
					Lower	Upper	
**Sex**			***				
Male (ref.)	1643	89.2		1			
Female	1770	92.4		1.6	1.3	2.1	***
**Age**			***				
15–24 years (ref.)	495	85.8		1			
25–34 years	571	90.5		1.5	1.0	2.1	*
35–44 years	598	91.1		1.6	1.1	2.3	*
45–54 years	611	92.0		2.0	1.4	2.9	***
55–64 years	550	91.9		1.9	1.3	2.9	***
65–74 years	341	92.9		2.0	1.3	3.4	**
75–85 years	247	93.7		2.5	1.4	4.4	**
**Monthly income**			***				
€0–1100 (ref.)	1038	87.4		1			
€1101–1800	1219	93.5		1.8	1.4	2.5	***
>€1800	872	92.5		1.5	1.1	2.1	*
Don’t know/Declined to answer	284	88.0		1.0	0.7	1.5	>0.05
**Region**			**				
Paris region (ref.)	632	88.5		1			
North	210	88.3		1.1	1.0	2.7	*
Eastern Paris basin	260	88.1		1.0	0.7	1.7	>0.05
Western Paris basin	302	88.6		1.1	0.7	1.6	>0.05
West	485	93.8		2.1	0.7	1.7	>0.05
South-West	385	93.5		2.1	1.4	3.2	***
Mediterranean basin	418	91.5		1.5	1.3	3.4	**
Center-East	420	91.6		1.5	1.0	2.3	>0.05
East	301	92.5		1.7	1.0	2.3	>0.05
**Education level**			***				
<High school	1770	88.9		1			
High school	672	93.7		2.1	1.5	3.0	***
University level	971	92.6		1.5	1.1	2.1	**

Legend: OR = odds ratio; CI = confidence interval; ref. = reference; * = *p* <0.05, ** = *p* <0.01, *** = *p* <0.001. Note: For this analysis, data from 3413 of the 3764 could be included due to missing data.

**Table 4:** Participant perceptions of nutritional factors and cancer risk in 2015.

Questions and Responses	*n*	%	*n*	%	*n*	%	*n*	%
**In Your Opinion, Frequent Consumption of (a) (b) (*n* = 3764)**								
	(b) can lower cancer risk		(b) can increase cancer risk		(b) has no influence		(b) I don’t know	
(a) Fruit and vegetables	**2187**	**58.1**	145	3.9	1111	29.5	321	8.5
(a) Red meat	234	6.2	**1602**	**42.6**	1260	33.5	669	17.8
(a) Milk	**443**	**11.8**	503	13.4	2009	53.4	809	21.5
(a) Cold meats	89	2.4	**2342**	**62.2**	801	21.3	533	14.2
(a) White meat	743	19.7	180	4.8	2157	57.3	684	18.2
(a) Fish	1442	38.3	293	7.8	1484	39.4	545	14.5
(a) Salt and salty foods	145	3.8	**2053**	**54.6**	965	25.6	601	16.0
(a) Potatoes	278	7.4	138	3.7	2434	64.7	914	24.3
**In your opinion, regular physical activity (b) (*n* = 3764)**								
	**2636**	**70.0**	49	1.3	743	19.7	335	8.9
**In your opinion, overweight or obesity (b) (*n* = 3764)**								
	80	2.1	**2843**	**75.5**	443	11.8	398	10.6
**In your opinion, breastfeeding (b) the mothers’ breast cancer risk (for women only, *n* = 1918)**								
	**653**	**34.0**	77	4.0	724	37.7	464	24.2

Legend: in bold type = factors known to increase or lower cancer development [3].

**Table 5:** Sociodemographic factors associated with perception of the role of food in cancer risk (*n* = 3762).

Variables	Model 1: Fruit and Vegetables		Model 2: Red Meat		Model 3: Cold Meat		Model 4: Salt and Salty Foods	
	OR (95% CI)	*p*-Value	OR (95% CI)	*p*-Value	OR (95% CI)	*p-Value*	OR (95% CI)	*p*-Value
**Sex**								
Male (ref.)	1		1		1		1	
Female	1.1 (0.9–1.3)	>0.05	1.1 (1.0–1.3)	>0.05	1.0 (0.9–1.2)	>0.05	0.9 (0.7–1.0)	*
**Age**								
15–24 years (ref.)	1		1		1		1	
25–34 years	0.9 (0.7–1.1)	>0.05	1.0 (0.8–1.2)	>0.05	1.1 (0.9–1.4)	>0.05	0.7 (0.6–0.9)	*
35–44 years	0.9 (0.7–1.2)	>0.05	1.4 (1.1–1.8)	**	1.2 (0.6–1.5)	>0.05	0.6 (0.5–0.7)	***
45–54 years	1.5 (1.2–1.8)	**	1.5 (1.2–1.9)	**	1.4 (1.1–1.8)	**	0.4 (0.4–0.6)	***
55–64 years	1.3 (1.0–1.7)	*	1.8 (1.4–2.2)	***	1.4 (1.1–1.8)	**	0.5 (0.4–0.6)	***
65–74 years	1.2 (0.9–1.5)	>0.05	1.8 (1.4–2.4)	***	1.3 (1.0–1.7)	>0.05	0.4 (0.3–0.6)	***
75–85 years	0.9 (0.7–1.3)	>0.05	1.3 (0.9–1.7)	>0.05	1.1 (0.8–1.5)	>0.05	0.3 (0.2–0.4)	***
**Monthly income**								
*€*0–1100 (ref.)	1		1		1		1	
*€*1101–1800	1.3 (1.1–1.5)	**	1.5 (1.3–1.8)	***	1.3 (1.1–1.5)	**	1.4 (1.1–1.6)	***
>€1800	1.1 (0.9–1.4)	>0.05	1.6 (1.3–2.0)	***	1.3 (1.0–1.6)	*	1.3 (1.1–1.6)	*
Don’t know/Declined to answer	0.8 (0.7–1.1)	>0.05	1.2 (0.9–1.6)	>0.05	0.8 (0.6–1.0)	>0.05	0.9 (0.7–1.2)	>0.05
**Education level**								
<High school	1		1		1		1	
High school	1.5 (1.2–1.8)	***	1.1 (0.9–1.4)	>0.05	1.1 (1.0–1.4)	>0.05	0.9 (0.7–1.0)	>0.05
University level	2.0 (1.6–2.4)	***	1.7 (1.4–2.1)	***	1.5 (1.3–1.9)	***	1.2 (1.0–1.5)	>0.05
**Occupation ^1^**								
Employee (ref.)	1		1		1		1	
Laborer	1.2 (1.0–1.5)	*	1.1 (0.9–1.4)	>0.05	0.8 (0.7–1.0)	*	1.0 (0.8–1.5)	>0.05
Tradesperson, storekeeper, self-employed worker, or farmer	1.1 (0.8–1.5)	>0.05	1.1 (0.8–1.4)	>0.05	0.7 (0.6–1.0)	*	1.1 (0.8–1.5)	>0.05
Intermediate occupations	1.6 (1.3–2.0)	***	1.2 (0.9–1.4)	>0.05	0.9 (0.7–1.1)	>0.05	1.1 (0.9–1.3)	>0.05
Higher occupations	1.6 (1.2–2.0)	***	1.2 (0.9–1.5)	>0.05	0.9 (0.7–1.1)	>0.05	1.0 (0.8–1.3)	>0.05
Other non-workers	1.2 (0.8–1.6)	>0.05	1.2 (0.8–1.6)	>0.05	0.8 (0.6–1.1)	>0.05	0.7 (0.5–0.9)	*
**Perceived level of information on the health effects of diet**								
Very or somewhat poorly informed (ref.)	1		1		1		1	
Very or somewhat well informed	1.8 (1.6–2.1)	***	1.4 (1.2–1.6)	***	1.9 (1.7–2.2)		1.7 (1.5–2.0)	***

Legend: OR = odds ratio; CI = confidence interval; ref. = reference; * = *p* < 0.05, ** = *p* < 0.01, *** = *p* < 0.001; ^1^ The occupations listed are commonly used in France. “Employee” refers to employees with limited responsibilities such as administrative employees, or staff in direct contact with customers. “Laborer” refers to workers at a factory, drivers, or handling workers. “Intermediate occupations” refers to employees with intermediate responsibilities such as nurses, schoolteachers or social workers. “Higher occupations” refers to employees with senior responsibilities, such as professors, engineers, physicians or department directors.

**Table 6:** Physical activity (*n* = 3764), overweight and obesity (*n* = 3762), and breastfeeding (*n* = 1918 women) as factors associated with cancer risk.

Variables	Model 5: Physical Activity		Model 6: Overweight and Obesity		Model 7: Breastfeeding	
	OR (95% CI)	*p*-Value	OR (95% CI)	*p*-Value	OR (95% CI)	*p*-Value
**Sex**						
Male (ref.)	1		1		/	/
Female	0.8 (0.7–0.9)	***	0.7 (0.6–0.9)	***	/	/
**Age**						
15–24 years (ref.)	1		1		1	
25–34 years	0.7 (0.5–0,9)	**	0.6 (0.5–0.6)	**	1.7 (1.2–2.4)	**
35–44 years	0.6 (0.4–0.7)	***	0.5 (0.3–0.6)	***	1.6 (1.1–2.4)	**
45–54 years	0.6 (0.5–0.8)	***	0.5 (0.4–0.7)	***	2.0 (1.4–2.9)	***
55–64 years	0.6 (0.5–0.9)	**	0.7 (0.5–0.9)	**	1.6 (1.1–2.4)	*
65–74 years	0.6 (0.4–0.8)	**	0.6 (0.4–0.8)	**	1.6 (1.0–2.4)	*
75–85 years	0.7 (0.5–0.9)	*	0.4 (0.3–0.6)	***	1.9 (1.2–2.9)	**
**Monthly income**						
€0–1100 (ref.)	1		1		1	
€1101–1800	1.3 (1.1–1.7)	**	1.3 (1.0–1.5)	*	0.9 (0.7–1.1)	>.05
>€1800	1.2 (0.9–1.5)	>.05	1.2 (1.0–1.6)	>.05	1.0 (0.7–1.3)	>.05
Don’t know/Declined to answer	0.8 (0.6–1.0)	>.05	0.9 (0.7–1.2)	>.05	0.5 (0.3–0.8)	**
**Education level**						
<High school	1		1		1	
High school	1.7 (1.4–2.2)	***	1.4 (1.1–1.7)	**	1.6 (1.2–2.1)	**
University level	1.9 (1.6–2.4)	***	1.6 (1.2–2.0)	***	1.8 (1.3–2.4)	***
**Occupation ^1^**						
Employee (ref.)	1		1		1	
Laborer	0.7 (0.6–0.9)	**	1.1 (0.9–1.4)	>0.05	1.4 (1.1–1.9)	*
Tradesperson, storekeeper, self-employed worker and farmer	0.8 (0.6–1.0)	>0.05	0.8 (0.6–1.1)	>0.05	1.0 (0.7–1.5)	>0.05
Intermediate occupations	1.0 (0.8–1.3)	>0.05	1.3 (1.0–1.7)	*	1.2 (0.9–1.6)	>0.05
Higher occupations	1.2 (0.9–1.6)	>0.05	1.2 (0.9–1.5)	>0.05	1.1 (0.8–1.5)	>0.05
Other non-workers	0.5 (0.3–0.6)	***	0.8 (0.6–1.1)	>0.05	0.5 (0.3–0.8)	**
**Level of perceived information on the health effects of diet**						
Very or somewhat poorly informed (ref.)	/	/	1		/	/
Very or somewhat well informed	/	/	1.6 (1.4–1.9)	***	/	/
**Level of perceived information on the health effects of physical activity**						
Very or somewhat poorly informed (ref.)	1		1		/	/
Very or somewhat well informed	2.0 (1.7–2.3)	***	1.3 (1.1–1.6)	**		

Legend: OR = odds ratio; CI = confidence interval; ref. = reference; * = *p* < 0.05, ** = *p* < 0.01, *** = *p* <0.001; ^1^ The occupations listed are commonly used in France. “Employee” refers to employees with limited responsibilities such as administrative employees, or staff in direct contact with customers. “Laborer” refers to workers at a factory, drivers, or handling workers. “Intermediate occupations” refers to employees with intermediate responsibilities, such as nurses, schoolteachers, or social workers. “Higher occupations” refers to employees with senior responsibilities, such as professors, engineers, physicians, or department directors.

**Table 7:** Evolution of participant perceptions of nutritional factors: perception rates in 2010 and 2015.

**Response**	**Fruit and Vegetables**		**Diff.**	***p*-Value for χ^2^ Test**	**Red Meat**		**Diff.**	***p*-Value for χ^2^ Test**
	**2010**	**2015**		***	**2010**	**2015**		***
Lowers	**56.9**	**58.4**	**+1.5**		4.1	6.2	+2.1	
Increases	2.7	3.9	+1.2		**31.0**	**42.7**	**+11.7**	
Has no influence on cancer risk	17.9	29.7	+11.8		16.1	33.8	+17.7	
Don’t know	22.5	8	−14.5		48.9	17.3	−31.6	
**Response**	**Cold meats**		**Diff.**	***p*-Value for χ^2^ test**	**Salt and salty foods**		**Diff.**	***p*-Value for χ^2^ test**
	**2010**	**2015**		***	**2010**	**2015**		***
Lowers	2.3	2.3	0		3.0	3.6	+0.6	
Increases	**47.9**	**62.3**	**+14.4**		**37.1**	**55.6**	**+18.5**	
Has no influence on cancer risk	8.2	21.5	+13.3		12.4	25.2	+12.8	
Don’t know	41.6	13.9	−27.7		47.5	15.6	−31.9	
**Response**	**Milk**		**Diff.**	***p*-Value for χ^2^ test**	**White meat**		**Diff.**	***p*-Value for χ^2^ test**
	**2010**	**2015**		***	**2010**	**2015**	***	
Lowers	7.6	11.9	+4.3		13.5	20.0	+6.5	
Increases	5.2	13.9	+8.7		2.5	4.9	+2.4	
Has no influence on cancer risk	30.1	52.9	+22.8		32.5	57.3	+24.8	
Don’t know	57.2	21.3	−35.9		51.5	17.9	−33.6	
**Response**	**Fish**		**Diff.**	***p*-Value for χ^2^ test**	**Potatoes**		**Diff.**	***p*-Value for χ^2^ test**
	**2010**	**2015**		***	**2010**	**2015**		***
Lowers	38.1	38.7	+0.6		4.0	7.3	+3.3	
Increases	3.3	7.8	+4.5		2.2	3.8	+1.6	
Has no influence on cancer risk	21.4	39.3	+17.9		34.8	64.5	+29.7	
Don’t know	37.2	14.2	−23		59.1	24.3	−34.8	
**Response**	**Physical Activity**		**Diff.**	***p*-Value for χ2 Test**	**Overweight and Obesity**		**Diff.**	***p*-Value for χ2 Test**
	**2010**	**2015**		***	**2010**	**2015**		***
Lowers	**59.4**	**70.3**	**+10.9**		3.3	2.0	−1.3	
Increases	1.9	1.4	−0.5		**63.9**	**76.0**	**+12.1**	
Has no influence on cancer risk	19.4	19.6	+0.2		9.4	11.5	+21.1	
Don’t know	19.3	8.7	−10.6		23.4	10.5	−12.9	
**Response**	**Breastfeeding**		**Diff.**	***p*-Value for χ2 Test**				
	**2010**	**2015**		***				
Lowers	**24.4**	**34.4**	**+10**					
Increases	2.9	4.2	+1.3					
Has no influence on cancer risk	27.4	37.9	+10.5					
Don’t know	45.4	23.5	−21.9					

Legend: Diff. = Difference observed between 2015 and 2010; *** = *p* <0.001; in bold type = factors known to increase or lower cancer development [3].
